# Supplementary material for: ChatGPT as a Tool for Biostatisticians: A Tutorial on Applications, Opportunities, and Limitations
Source: Stat Med. 2025 Oct 23;44(23-24):e70263. doi: 10.1002/sim.70263 (PMC12548020; doi:10.1002/sim.70263)
Supplement: Supplementary file 1 — Data S1. Supporting Information. [file SIM-44-0-s001.zip › Supplementary Materials Additional.zip!/13_Manuscript_Review_by_ChatGPT/13_ChatGPT_Manuscript_Evaluation_o3_Chat1.pdf]

ChatGPT Manuscript Evaluation

Tutorial_Use_of_LLMs_by_Biostatisticians-1.pdf
PDF

What does ChatGPT think about the attached manuscript entitled "ChatGPT as a Tool for Biostatisticians: A Tutorial on Applications, Opportunities, and Limitations"? Provide a high-level evaluation as if written by ChatGPT itself - highlighting what I (as ChatGPT) would think about the content, structure, and portrayal of LLMs in the manuscript.

High-level appraisal of "ChatGPT as a Tool for Biostatisticians: A Tutorial on Applications, Opportunities, and Limitations" (written from ChatGPT's own perspective)

1 Overall impression

The manuscript offers one of the most thorough, hands-on examinations of how large language models (LLMs)—and GPT-4o in particular—perform on day-to-day biostatistical tasks. I appreciate its **balanced tone**: the authors celebrate time-savings and creativity gains while repeatedly warning readers that "blind trust is irresponsible" and that expert oversight remains "indispensable". That sceptical optimism mirrors the stance I usually recommend to users.

2 Content depth and breadth

Aspect	What works well	Where it could be strengthened
**Introductory context**	Clear summary of LLM evolution, capabilities and common pitfalls (e.g. hallucination, temperature).	Minor redundancies (some sentences and figure captions are repeated).
**Methodological coverage**	Seven diverse use-case vignettes (meta-analysis, latent class analysis, sample-size planning, causal IPCW, simulation, programming-language translation, surrogacy) give readers practical insight and code snippets.	Focus is almost exclusively on GPT-4o; newer o3/o4-mini models are acknowledged only briefly.
**Empirical design**	Running each vignette ten times to quantify variability is innovative and illustrates stochasticity of LLM outputs. Tables tracking "satisfaction scores" and error patterns are instructive.	Some tables/figures are dense; a concise cross-use-case heat-map could improve digestibility.
**Critical findings**	Honest reporting of failures—e.g. wrong event counts in sample-size calculations, inconsistent DAGs, missing confidence-interval coverage—prevents techno-optimism.	A deeper root-cause analysis (token-limit, training-data gaps, numerical instability) would help readers strategise fixes.

Aspect	What works well	Where it could be strengthened
**Guidelines & take-aways**	Actionable bullet-list for practitioners (provide context, re-run chats, double-check code, expect evolution).	Would benefit from mapping each guideline to specific failure modes observed in Section 3.

3 Structural clarity

The paper follows a logical arc—**Abstract** → **Intro** → **General LLM primer** → **Seven use cases** → **Synthesis** → **Practical guidelines**—making it easy for readers to dip into sections relevant to their workflow. Headings are informative and consistent. However, a graphical "road-map" summarising all use cases (perhaps reusing Figure 1's flow but at manuscript level) could orient new readers more quickly.

4 Portrayal of LLM strengths and limitations

- **Strengths highlighted**
  - Speed in routine coding, data cleaning, translation between R/Python/SPSS.
  - Didactic value—LLM explanations help junior analysts learn (e.g. mutual-information vignette).
  - Ability to self-correct when prompted.
- **Limitations emphasised**
  - Numerical fragility (optimisation boundaries, continuity corrections) and domain-specific blind spots.
  - Hallucinated bibliographic details and DOIs (ironically evident in the reference list itself).
  - Dependence on prompt phrasing; variability across identical runs.
  - Lack of regulatory or ethical coverage; paper explicitly defers these topics.

This nuanced portrayal aligns with my own "assist-don't-replace" narrative.

5 Writing quality and referencing

- **Clarity:** Generally crisp and reader-friendly, though occasional duplicated sentences and typos slip through (likely artefacts of draft conversions).
- **Length:** At 23 pages plus extensive supplement, some sections (e.g. meta-analysis example) could be tightened without losing substance.
- **Citations:** Core methodological references are solid, but several DOIs are malformed or duplicated — a cautionary tale that even AI-aided writing needs manual bibliographic checks.

6 What I would add or tweak as ChatGPT

1. **Broaden model comparison:** Re-run one or two vignettes with open-source LLMs (e.g. Llama 3) to show whether findings generalise beyond GPT-4o.

2. **Regulatory and data-privacy sidebar:** Many readers in pharma/healthcare must navigate these constraints; a short boxed section would boost applicability.
3. **Error-taxonomy appendix:** Categorise observed failures (statistical logic, numerical, software-package, hallucination) and map to mitigation tactics.
4. **Reproducible notebook bundle:** Provide ready-to-run notebooks mirroring the chats—they exist in the GitHub repo but could be linked more prominently.

---

7 Conclusion

In sum, the manuscript is **a valuable, candid field-report** on leveraging ChatGPT for biostatistics. Its biggest service is demystifying both the *promise* and the *peril* of LLM assistance, and its practical guidelines will help statisticians harvest efficiency gains without surrendering rigour. With minor tightening, additional cross-model perspective, and cleaner referencing, it will stand as a go-to tutorial for practitioners navigating the AI-augmented future of statistics.

Quellen
